# Risks of empiric glucocorticoid administration in elderly patients with inflammation of unknown origin: A case report

**DOI:** 10.1097/MD.0000000000042234

**Published:** 2025-04-18

**Authors:** Takashi Yamane, Chiharu Miyamoto

**Affiliations:** a Department of Rheumatology, Kakogawa Central City Hospital, Kakogawa, Japan.

**Keywords:** case report, gastric cancer, glucocorticoid, inflammation of unknown origin, venous thrombosis embolism

## Abstract

**Rationale:**

Inflammation of unknown origin (IUO) in elderly patients is frequently caused by noninfectious inflammatory diseases. When infections and malignancies are ruled out, glucocorticoids (GCs) are often administered as an empirical diagnostic treatment. However, GC carries risks, including osteoporosis and venous thromboembolism (VTE), and in cases of undiagnosed malignancies, GC use may delay definitive diagnosis. Additionally, VTE itself can mimic IUO by inducing inflammation, making diagnosis more complex when multiple conditions coexist. Despite these concerns, comprehensive studies on the risks of empirical GC treatment for IUO are lacking. This case highlights these potential risks.

**Patient concerns:**

An 84-year-old Japanese woman with no prior medical history presented with a 5-month history of fever and anorexia without identifiable causes. She exhibited persistently elevated C-reactive protein levels, and initial antimicrobial therapy was ineffective.

**Diagnoses:**

The patient was initially suspected of having noninfectious inflammatory diseases. However, after experiencing complications from GC therapy, further investigations revealed Stage I gastric adenocarcinoma.

**Interventions:**

Two weeks after hospital admission, prednisolone 30 mg/day was initiated for suspected noninfectious inflammatory diseases, leading to normalization of C-reactive protein. However, upon GC reduction, the inflammatory markers increased again, necessitating continued prednisolone administration. The patient subsequently developed a compression fracture and was later readmitted with right leg edema and pain. Imaging revealed VTE, likely resulting from GC use and immobilization from the fracture. Anticoagulation therapy was initiated, and GC tapering was performed. Despite persistent inflammation, further diagnostic evaluations, including F-fluorodeoxyglucose positron emission tomography/computed tomography, revealed hyperaccumulation in the stomach, leading to endoscopic confirmation of Stage I gastric adenocarcinoma. The patient underwent laparoscopy-assisted distal gastrectomy.

**Outcomes:**

One year after surgery, no recurrence of malignancy was observed. The patient’s inflammatory markers normalized, and no further thromboembolic events were observed.

**Lessons:**

This case demonstrates that GC therapy in elderly IUO patients can lead to severe complications, including VTE, and delayed malignancy diagnosis. Thorough malignancy and thrombus screening should be conducted before GC initiation. Additionally, when VTE occurs in IUO patients, malignancy should be reassessed, even if other risk factors are present. This case underscores the importance of caution when considering empirical GC therapy for IUO.

## 1. Introduction

In a study of 256 cases of classical fever of unknown origin (FUO) in Japanese adults, noninfectious inflammatory diseases (NIID) and cancers were more common in patients over 65 years of age, with their frequency increasing. Meanwhile, the proportion of patients whose cause remains unidentified is also rising, with >20% of NIID cases falling into this category.^[1]^ Since NIID includes diseases for which glucocorticoid (GC) may be effective, like adult-onset Still’s disease, vasculitis, polymyalgia rheumatica (PMR),^[2]^ it is expected that in real world clinical practice, GC would be initiated as a diagnostic treatment once malignancy is considered excluded. However, a recent study from Finland found that in 9.3% of patients initially diagnosed with PMR, actual diagnosis was malignancies,^[3]^ and a report from Spain also found that approximately 10% of patients presenting with PMR-like symptoms actually had a different disease, most often cancer.^[4]^ If GC is administered to a patient with malignancy, its anti-inflammatory effect can delay the correct diagnosis. Moreover, GC administration is also associated with adverse events, such as GC induced osteoporosis and venous thrombosis embolism (VTE), which affect mortality and quality of life. Pathological fractures due to GC induced osteoporosis can lead to immobility and trigger VTE, and GCs themselves also carry a risk of VTE.^[5–7]^ In a report from the Netherlands, 4495 patients with pulmonary embolism during GC treatment, the risk was reported to be twice as high as in patients not receiving GC, even at doses as low as 5 mg of prednisolone (PSL) equivalent per day.^[8]^ On the other hand, VTE itself can also be a cause of fever and inflammation of unknown origin (IUO) because various chemokines and cytokines are released by the thrombus,^[9]^ further complicating the diagnosis when malignancies – both a cause of FUO/IUO and a risk factor for VTE – are present.^[4]^

Here we report a case of an elderly Japanese patient who developed persistent inflammation despite GC treatment for IUO. Although extensive VTE was identified, it was initially thought to be the cause of inflammation; however, the condition did not improve with anticoagulation and taper off the GC.

Given the lack of comprehensive research on the risks of empirical GC use in IUO, we present this case to highlight the potential complications of this treatment approach.

## 2. Material and methods

An 84-year-old Japanese woman with no underlying diseases visited a hospital due to fever and anorexia persisting for 5 months, with no apparent triggers. Blood examination revealed an elevated C-reactive protein (CRP) level of 16.5 mg/dL, and she was admitted. Blood and urine cultures were negative, and a plain computed tomography (CT) scan did not reveal a definitive cause. Suspecting a bacterial infection, antimicrobial therapy was initiated, but the patient’s condition did not improve. 2 weeks later, PSL at 30 mg per day was started for NIID, and the CRP level became negative. However, after reducing the PSL dose, the CRP level increased again, so she was continued on 20 mg of daily PSL.

## 3. Results and discussion

One month later, she developed back pain and was diagnosed with an osteoporotic vertebral compression fracture. She was discharged after 2 months, but at the time of discharge, she developed edema in her right lower leg and was unable to walk due to pain extending from her right thigh to the lumbar region. 1 month after discharge, she was re-hospitalized. Dual-energy X-ray absorptiometry showed a young adult mean of 48% for the lumbar spine and 43% for the thigh, and a corset was fitted. The patient was referred to our hospital at the request of her family. Physical examination revealed bilateral lower leg edema and mild chest pain, blood tests showed elevated inflammatory markers and abnormal coagulation and fibrinolysis (Table 1).

**Table 1:** Laboratory findings at initial presentation.

Parameter	Result	Normal range	Parameter	Result	Normal range
WBC (×10^3^/μL)	6.87	3.3 to 8.6	TC (mg/dL)	196	142 to 248
RBC (×10^6^/μL)	4.58	3.86 to 4.92	TG (mg/dL)	63	30 to 117
Hb (g/dL)	13.5	11.6 to 14.8	HDL-C (mg/dL)	74	48 to 103
PLT (×10^3^/μL)	263	158 to 348	LDL-C (mg/dL)	109	65 to 163
PT (%)	98.8	70 to 130	T-Bil (mg/dL)	1.1	0.4 to 1.5
APTT	26.6	26 to 38	BUN (mg/dL)	19.7	8 to 20
D-dimer	13.6	<1.0	Cre (mg/dL)	0.67	0.46 to 0.79
Urinalysis	Normal	–	UA (mg/dL)	3.5	2.6 to 5.5
			Ca (mg/dL)	9	8.8 to 10.1
TP (g/dL)	6.2	6.6 to 8.1	Na (mEq/L)	134	138 to 145
Alb (g/dL)	3.4	4.1 to 5.1	K (mEq/L)	4.4	3.6 to 4.8
AST (U/L)	28	13 to 30	Cl (mEq/L)	100	101 to 108
ALT (U/L)	10	7 to 23	Glu (mg/dL)	137	73 to 109
LD (IFCC, U/L)	333	124 to 222	HbA1c (%)	6.0	4.9 to 6.0
ALP (IFCC, U/L)	127	38 to 113	C3 (mg/dL)	133	73 to 138
γGT (U/L)	18	9 to 32	CRP (mg/dL)	4.6	0 to 0.14
CK (U/L)	96	41 to 153	RF (IU/mL)	227	<20
Lipase (U/L)	29	13 to 53	Antinuclear Antibody (ANA)	<40	<40
			Anti-CCP Antibody (IU/mL)	<0.5	<4.5
			P-ANCA	(–)	–
			C-ANCA	(–)	–

Alb = albumin, ALP = alkaline phosphatase, ALT = alanine aminotransferase, ANCA = anti-neutrophil cytoplasmic antibodies, AST = aspartate aminotransferase, BUN = blood urea nitrogen, CCP = cyclic citrullinated peptide, CK = creatine kinase, Cre = creatinine, Glu = glucose, HbA1c = hemoglobin A1c, HDL-C = high-density lipoprotein cholesterol, LD = lactate dehydrogenase, LDL-C = low-density lipoprotein cholesterol, RF = rheumatoid factor, T-Bil = total bilirubin, TC = total cholesterol, TG = triglycerides, TP = total protein, UA = uric acid, γGT = gamma-glutamyl transferase.

**Figure 1.**
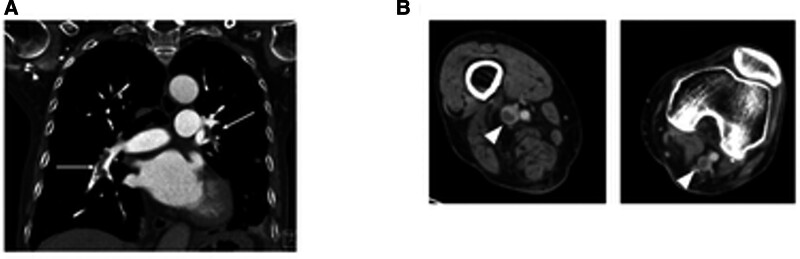
Contrast-enhanced computed tomography of venous thrombosis embolism. (A) Partial filling defect in the bilateral pulmonary arteries indicating pulmonary embolism (arrow). (B) Deep venous thrombosis in the bilateral popliteal veins (arrowhead).

**Figure 2.**
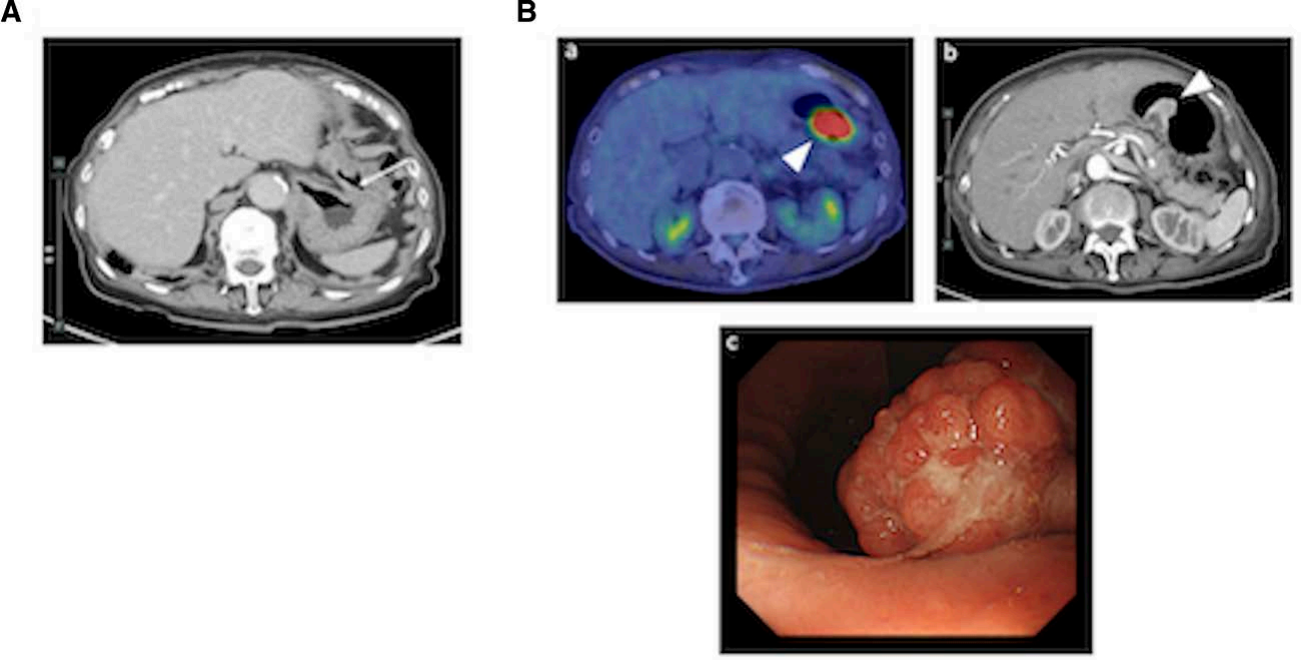
Imaging findings in gastric cancer during the clinical course. (A) Tumor in the angular incisure (arrow) is not evident on plain CT at the initial examination. (B) a. FDG-PET/CT performed 6 months later shows strong accumulation in the stomach. b. Contrast-enhanced CT reveals an elevated lesion consistent with the accumulation. c. Upper endoscopy shows advanced gastric cancer in the angular incisure, and biopsy confirms a diagnosis of adenocarcinoma.

A contrast enhanced CT scan revealed pulmonary embolism and deep vein thrombosis (DVT), but there was no evidence of cancer or other diseases (Fig. 1). Antinuclear antibodies, lupus anticoagulant, and anticardiolipin antibodies were all negative. Immobility due to the compression fracture and GC administration were diagnosed as the main causes of VTE, and apixaban was started. GCs were tapered off to confirm the diagnosis of IUO. Although GC was discontinued in April 2023, and also the thrombus resolved, the CRP level did not return to normal, and no abnormal findings leading to a diagnosis were detected. Therefore, (18)F-fluorodeoxyglucose position emission tomography/CT was performed to investigate the unknown inflammation considering the possibility of large vasculitis. Hyperaccumulation was observed in the stomach. Upper endoscopy revealed advanced gastric cancer in the gastric angle, and histopathology confirmed adenocarcinoma. Contrast-enhanced CT showed no lymph node metastasis or distant metastasis, and the patient was diagnosed with Stage I gastric cancer and underwent laparoscopy-assisted distal gastrectomy (Fig. 2).

**Figure 3.**
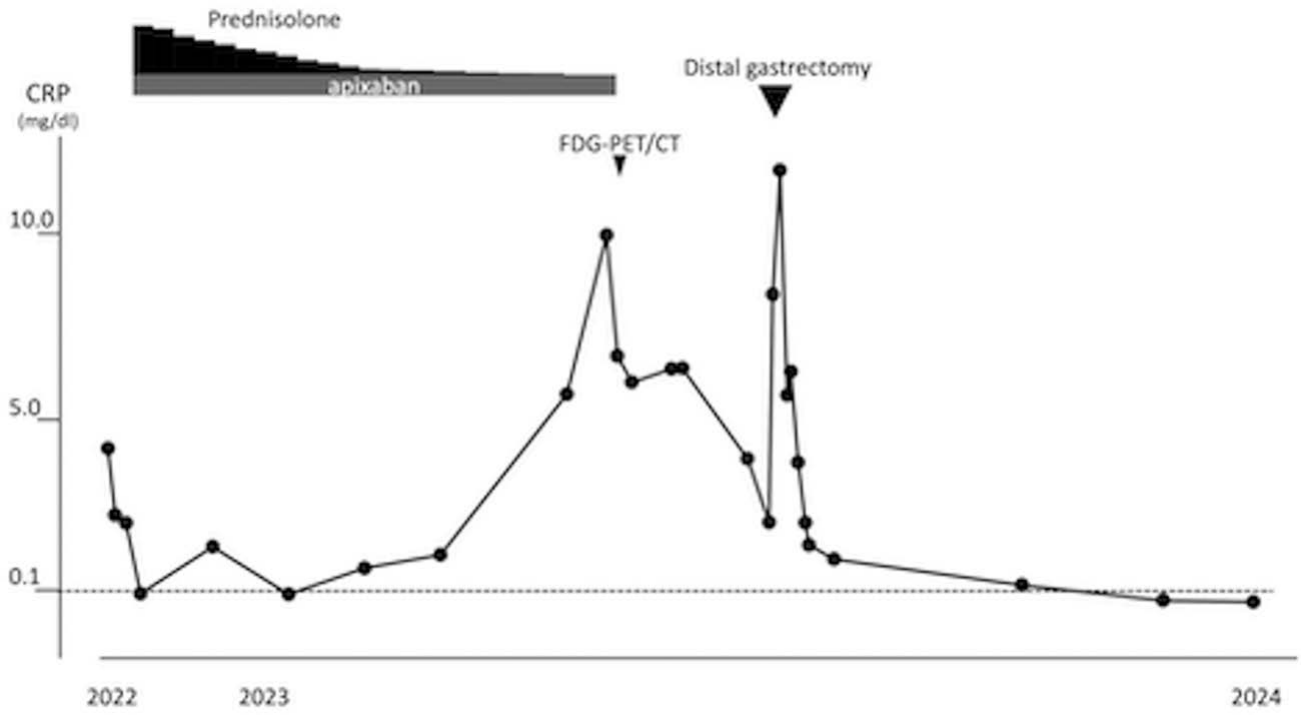
Clinical course to achieve CRP negativity. Dotted line indicates normal range. CRP = C-reactive protein; FDG-PET/CT = fluorodeoxyglucose position emission tomography/CT.

**Figure 4.**
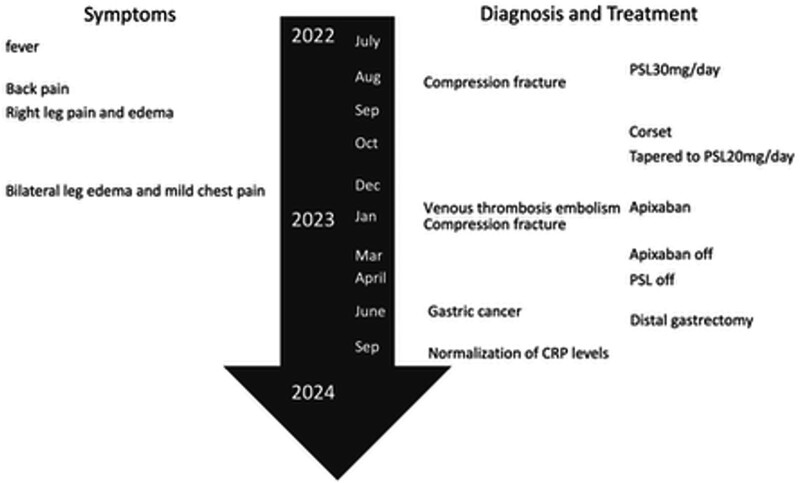
Time course of the case. CRP= C-reactive protein, PSL = prednisolone.

Following surgery, no recurrence of malignancy or elevation in CRP was observed 1 year postoperatively. The patient’s inflammatory markers normalized, and no further thromboembolic events occurred (Figs. 3 and 4).

As a study that performed lower extremity venous Doppler ultrasonography to search for the cause of IUO reported, 6.7% of patients were diagnosed with DVT, this suggest that DVT is a not uncommon cause of IUO.^[10]^ Since it is often difficult to diagnose DVT based on symptoms or scoring systems alone,^[11–13]^ imaging studies are essential. In particular, contrast-enhanced CT is useful to examining areas difficult to assess by ultrasound, such as the inferior vena cava and iliac vein regions.^[14]^

The incidence of DVT following spinal compression fractures has been reported as 0.9% in a study of approximately 40,000 cases,^[15]^ while a Japanese report noted an incidence of 12% in 50 cases.^[16]^ DVT should be considered as a cause of IUO following immobilization, not only cases of compression fractures. The recommended duration of anticoagulant therapy for VTE is 3 months,^[17]^ and in this case, thrombus resolution was confirmed after 3 months of treatment. However, CRP levels did not normalize, prompting further investigation for causes of IUO beyond DVT.

Among imaging modalities used for FUO/IUO diagnosis, PET/CT has been frequently reported as useful. A prospective study of 240 FUO/IUO cases reported that PET/CT led to a diagnosis in 79% of cases,^[2]^ and it is particularly effective for inflammatory diseases, including rheumatologic diseases.^[18,19]^ In a single-center retrospective cohort study, PET/CT was significantly more sensitive than contrast-enhanced CT.^[20]^ In our case, PET/CT was performed by considering the possibility of NIID without clinical symptoms such as large vessel vasculitis as a differential. However, gastric cancer was ultimately diagnosed.

Since CRP is considered an indicator of disease activity in solid tumors, including gastric cancer,^[21,22]^ we concluded the gastric cancer was the underlying cause of the persistent inflammation from the beginning. Notably, despite the resolution of thrombus with anticoagulation and GC tapering, CRP levels remained elevated, prompting further diagnostic evaluation. This highlights the importance of monitoring inflammatory markers even after apparent resolution of suspected causes, as persistent inflammation may indicate an undiagnosed malignancy.

Since contrast-enhanced CT initially showed no signs of gastric cancer at the outset, further examination was not performed, but the development of VTE might have been influenced by cancer-related hypercoagulability.^[23]^ Considering that 2.4% (n = 2354) of 116,048 VTE cases were associated with cancer diagnosed within 6 months and that approximately 60% of 216 patients with VTE who were diagnosed with cancer were in the early stages,^[24]^ clinicians should search for cancer when VTE is identified, even if other risk factors are present.

The usefulness of PET/CT in diagnosing DVT has been reported by the correlation of PET signal intensity with neutrophils in thrombus in a mouse model as well as its high diagnostic accuracy in prospective clinical trials.^[25,26]^ If healthcare costs are not a c consideration, PET/CT may be the optimal imaging modality for IUO, including DVT.

However, false negatives for early-stage cancer remain a concern with PET/CT.^[27]^ In Japanese data, only 47 of 124 cases of gastric cancer were detected by PET/CT.^[28]^ Therefore, each cancer type should be investigated using the recommended diagnostic methods.

Nevertheless, the primary issue in this case was that GCs were administered without a definitive diagnosis. Although there are no clear reports on the actual empiric administration of GC and the associated risks, it is likely that this is done in many cases in real clinical practice. As an example, although recent PMR guidelines advise against initiating GC treatment until the diagnosis is confirmed,^[29]^ the latest international study found that approximately 50% of PMR patients had been prescribed GC before being evaluated by a rheumatologist.^[30]^

This study is limited by being a single-case report, which restricts its generalizability. The absence of a control group prevents a direct assessment of the frequency and severity of risks associated with empirical GC administration in IUO.

## 4. Conclusion

This case demonstrated that empirical GC administration in an elderly IUO patient resulted in a compression fracture, secondary VTE, and delayed cancer diagnosis. The findings underscore the necessity of thorough malignancy and thrombus screening before initiating GC therapy in IUO. Additionally, persistent inflammation despite GC tapering and thrombus resolution should prompt further malignancy investigation. Given the lack of studies quantifying the risks of empirical GC use in IUO, this case highlights the need for cautious evaluation and further research on this treatment approach.

## Author contributions

**Conceptualization:** Takashi Yamane.

**Writing – original draft:** Takashi Yamane.

**Writing – review & editing:** Takashi Yamane, Chiharu Miyamoto.
